# Effects of age, seasonality, and reproductive status on the gut microbiome of Southern White Rhinoceros (*Ceratotherium simum simum*) at the North Carolina zoo

**DOI:** 10.1186/s42523-023-00249-5

**Published:** 2023-05-05

**Authors:** Christina M. Burnham, Erin A. McKenney, Kimberly Ange- van Heugten, Larry J. Minter, Shweta Trivedi

**Affiliations:** 1 Department of Animal Science, North Carolina State University, 120 W Broughton Dr, Raleigh, NC 27607 USA; 2 Department of Applied Ecology, North Carolina State University, 100 Brooks Ave, Raleigh, NC 27607 USA; 3 North Carolina Zoo, 4401 Zoo Parkway, Asheboro, NC 27205 USA

**Keywords:** Gut microbiome, Southern white rhinoceros, Temporal dynamics

## Abstract

**Background:**

Managed southern white rhinoceros (*Ceratotherium simum simum*) serve as assurance populations for wild conspecifics threatened by poaching and other anthropocentric effects, though many managed populations experience subfertility and reproductive failure. Gut microbiome and host health are inextricably linked, and reproductive outcomes in managed southern white rhinoceros may be mediated in part by their diet and gut microbial diversity. Thus, understanding microbial dynamics within managed populations may help improve conservation efforts. We characterized the taxonomic composition of the gut microbiome in the managed population of female southern white rhinoceros (n = 8) at the North Carolina Zoo and investigated the effects of seasonality (summer vs. winter) and age classes (juveniles (n = 2; 0–2 years), subadults (n = 2; 3–7 years), and adults (n = 4; >7 years)) on microbial richness and community structure. Collection of a fecal sample was attempted for each individual once per month from July-September 2020 and January-March 2021 resulting in a total of 41 samples analyzed. Microbial DNA was extracted and sequenced using the V3-V4 region of the 16S rRNA bacterial gene. Total operational taxonomic units (OTUs), alpha diversity (species richness, Shannon diversity), and beta diversity (Bray-Curtis dissimilarity, linear discriminant analysis effect size) indices were examined, and differentially enriched taxa were identified.

**Results:**

There were differences (p < 0.05) in alpha and beta diversity indices across individuals, age groups, and sampling months. Subadult females had higher levels of Shannon diversity (Wilcoxon, p < 0.05) compared to adult females and harbored a community cluster distinct from both juveniles and adults. Samples collected during winter months (January-March 2021) possessed higher species richness and statistically distinct communities compared to summer months (July-September 2020) (PERMANOVA, p < 0.05). Reproductively active (n = 2) and currently nonreproductive adult females (n = 2) harbored differentially enriched taxa, with the gut microbiome of nonreproductive females significantly enriched (p = 0.001) in unclassified members of *Mobiluncus*, a genus which possesses species associated with poor reproductive outcomes in other animal species when identified in the cervicovaginal microbiome.

**Conclusion:**

Together, our results increase the understanding of age and season related microbial variation in southern white rhinoceros at the North Carolina Zoo and have identified a potential microbial biomarker for reproductive concern within managed female southern white rhinoceros.

**Supplementary Information:**

The online version contains supplementary material available at 10.1186/s42523-023-00249-5.

## Background

The southern white rhinoceros (*Ceratotherium simum simum*) is a large grazing herbivore that leverages microbial fermentation in the hindgut to facilitate the digestion of complex plant carbohydrates [1]. These rhinoceros are a bastion of conservation success, having recovered from fewer than 100 individuals at the start of the 20th century to over 18,000 in 2017 [2]. Unfortunately, the wild population of southern white rhinoceros is once again declining due to a substantial increase in poaching and is considered near threatened by the International Union for Conservation of Nature [2, 3]. While managed southern white rhinoceros herds serve as assurance populations for imperiled wild conspecifics, many of the southern white rhinoceros females born in managed populations are either acyclic or cycle irregularly [4–6]. One proposed explanation for this trend is diet. In the past, managed diets of some rhinoceros contained substantially increased levels of phytoestrogens when compared to wild diets due to the reliance on alfalfa and other legume hays [6]. These phytoestrogens can interact with native mammalian estrogen receptors and have been negatively correlated with female southern white rhinoceros fertility [6, 7]. Some researchers have suggested that gut microbiota may affect rhinoceros reproductive outcomes by mediating phytoestrogen metabolism [7]. Beyond digestion and metabolism, the gut microbiome also mediates host immunity and has wide implications for host health [8–10]. Given these considerations, understanding gut microbial dynamics is crucial for the continued conservation and population management of southern white rhinoceros and other threatened species [11–13].

Gut microbiomes have been shown amongst numerous species to differ widely across individuals [14–16], driven by differences in gut morphology [17], feeding strategy [18, 19], age [20, 21], sex [22, 23], health status [24], and geographic location [25] of the host. The mammalian gut microbiome has also been shown to vary by season, with previous studies revealing marked seasonal changes in the community composition of humans [26], red squirrels (*Tamiasciurus hudsonicus*) [27], wood mice (*Apodemus sylvaticus)* [28], ground squirrels (*Ictidomys tridecemlineatus*) [29], giant pandas (*Ailuropoda melanoleuca*) [30], and horses (*Equus ferus caballus*)) [31].

Several researchers have attempted to characterize the gut microbiome of southern white rhinoceros [1, 7, 32, 33], though none have investigated differences in microbial composition due to season or age. We therefore aimed to determine the effects of seasonality and age on gut microbial community structure in a managed southern white rhinoceros population at the North Carolina Zoo. We also evaluated differences in microbial community composition between reproductively active and currently nonreproductive adult females. We hypothesized that North Carolina Zoo rhinoceros would display individual, age and seasonal differences in microbial alpha and beta diversity, as measured by species richness, Shannon diversity, and Bray-Curtis dissimilarity. We predicted that juveniles would host greater richness and inter-individual variation compared to subadults or adults. We also predicted that feces collected during summer sample collection months (July-September 2020) would contain higher microbial species diversity and possess a different community composition compared to winter sample collection months (January-March 2021), due to increased outdoor access and availability of grasses during the summer.

## Methods

### Sample population

Fecal samples were collected from a population of eight female southern white rhinoceros managed at the North Carolina Zoo in Asheboro, NC (35.6298° N, 79.7648° W). Age classes for southern white rhinoceros in this study were assigned based on a previously published age classification system [34], with modifications considering that managed populations of white rhinoceros experience earlier age transitions than their wild counterparts [5]. Specifically, individuals were designated as juveniles (0–2 years; n = 2), subadults (3–7 years; n = 2; defined as animals that have stopped nursing and have begun puberty [5]), or adults (> 7 years; n = 4). Three of the adult female rhinoceros were wild-caught over twenty years prior to this study (F2, F3, F4); all other individuals were born in a zoo. All adult females arrived at the North Carolina Zoo in 2007 and were exposed to three different bulls during their tenure there. Each bull was unproven and had sired no calves upon introduction to the females. One of these bulls was later diagnosed with aspermia, while another was with the females for a limited time before succumbing to colic. The third bull arrived at the North Carolina Zoo in 2014 and successfully sired calves. Among the female rhinos, F1 had never conceived or shown hormonal cycling during periodic blood hormone monitoring. F2 and F4 had calved at previous institutions but neither had produced a calf while at the North Carolina Zoo until 2018. F3 had calved at a previous institution, but not in the 15 years since arriving at the North Carolina Zoo. Periodic hormonal monitoring over the years indicated that this animal cycles normally and can conceive but does not carry the pregnancy to term. Both F1 and F4 received reproductive hormone injections (progesterone and estradiol) as part of an estrus synchronization protocol in March of 2017. Due to unexpected winter weather, only F4 was exposed to the male during the maximum receptive period. While F4 successfully calved the following year, the birth of the calf did not correspond to the timing of the estrus synchronization and exposure to the male. The ages, age classification, origin, group designator, reproductive status, and reproduction notes for each individual are summarized in Table 1.

The North Carolina Zoo population of rhinoceros was split into two social groups, which were housed separately during this study. One group consisted of two cow-calf pairs (F2, J1, F4, J2), while the other consisted of the four remaining females (F1, F3, S1, S2). No animals were treated with antibiotics or other medications during the study period.

**Table 1:** Summary of individual characteristics of n = 8 female southern white rhinoceros (*Ceratotherium simum simum*) at the North Carolina Zoo

Individual	Age (Years)	Age Classification	Origin	Group	Reproductive Status	Reproduction Notes
J1	1	Juvenile	Zoo	G1	N/A	Immature
J2	1	Juvenile	Zoo	G1	N/A	Immature
S1	3	Sub-adult	Zoo	G2	N/A	Immature
S2	3	Sub-adult	Zoo	G2	N/A	Immature
F1	15	Adult	Zoo	G2	Nonreproductive	Acyclic
F2	24*	Adult	Wild	G1	Reproductive	Cycling and previously calved
F3	29	Adult	Wild	G2	Nonreproductive	Cycling and conceives but aborts embryos
F4	33*	Adult	Wild	G1	Reproductive	Cycling and previously calved

*Age estimates for wild-caught individuals

N/A- Not applicable

### Diets

Animal diets were standardized but varied by season and age (Table 2). Year-round, the subadult and adult rhinoceros were provided with 1.36 kg of Mazuri® Wild Herbivore Diet Hi-Fiber (St. Louis, MO, USA) nutritionally complete pellet (WH pellets) daily, while the juveniles were offered 0.68 kg of WH pellets daily. During the summer, when the rhinoceros had access to outdoor paddocks, the animals consumed approximately one quarter of a bale of timothy hay (*Phleum pretense*) (4.5 kg) each. During the winter, sub-adult and adult rhinoceros received approximately one bale of timothy hay (18 kg) per animal daily. Rhinoceros also had access to the 16-hectare Watani Grasslands habitat during the winter, where fescue (*Festuca arundinacea*), annual ryegrass (*Lolium multiflorum*), and Bermuda (*Cynodon dactylon*) grasses were available for grazing. The nutrients within these grasses have been previously described [35]. Timothy cubes, orchard grass (*Dactylis glomerate*), and alfalfa hay (*Medicago sativa*) were all offered in rotation for training and enrichment; these supplementary feeds were offered at < 5% of the daily diet during summer and < 10% in the winter.

**Table 2:** Summary of diets for n = 8 female southern white rhinoceros (*Ceratotherium simum simum*) at the North Carolina Zoo^1^

	Summer Daily Diet (kg)		Winter Daily Diet (kg)	
Age Class	Mazuri® WH^2^ Hi-Fiber Pellet	Timothy Hay	Mazuri® WH^2^ Hi-Fiber Pellet	Timothy Hay
**Juvenile**	0.68	0	0.68	0
**Subadult**	1.36	4.5^3^	1.36	18.0^3^
**Adult**	1.36	4.5^3^	1.36	18.0^3^

^1^Pasture available *ad libitum*

^2^WH- Wild Herbivore (Mazuri**®**, St. Louis, MO, USA)

^3^Approximately

### Housing

Both social groups were housed in the rhinoceros barn, which consisted of a series of eight small stalls (37 m² each) and one maternity stall (52 m²). The cow-calf pair (F2, J1, F4, J2) group (denoted Group 1 or G1) was rotated routinely between either the maternity stall or a combination of two to three regular stalls. Both sets of stalls had adjoining outdoor paddocks of roughly 111 m², with asphalt and sand bed substrates. The remaining non-lactating and subadult female (F1, F3, S1, S2) group (denoted Group 2 or G2) had access to five adjoining stalls (for a total of 17 m² of space) when housed inside. The rhinoceros barn had forced air heating set at 10 °C, with no cooling ability.

During each day of the summer collection period (July-September 2020), one group of rhinoceros was rotated into a 2000 m² outdoor habitat known as a boma, while the other group was housed inside. The boma included a sand and rock paddock with some grass access adjacent to the 16-hectare Watani Grasslands habitat. No rhinoceros were allowed access to the habitat during the summer collection period (July-September 2020) due to a cyanobacterial algal bloom in the lake.

During winter collection period (January-March), Group 2 was allowed access to an outdoor holding area when temperatures were > 2 °C. Their outdoor holding comprised three adjoining paddocks totaling 1450 m², including a heated lean-to in the largest paddock. When outdoor access was restricted, the group was split into two pairs: one pair was held in 111 m² of combined stalls with access to approximately 150 m² of outdoor holding, while the other pair was held in 74 m² of combined stalls with access to 335 m² of outdoor holding. Individuals were rotated through different pairings, and pairs were rotated through different sets of stalls, so that each individual was equally exposed to all group members and indoor environments. Group 1 had access to the outdoor paddocks when the temperature was > 4.5 °C. Both groups also had access to the Watani Grasslands habitat in the winter when temperatures rose above 0 °C (G2) and 7 °C (G1).

### Sample collection, storage, and DNA extraction

Zoo staff attempted to collect a fecal sample from each rhinoceros within 30 min of defecation once per month during the months of July-September in 2020 and January-March in 2021. Staff collected the samples between days 20 and 30 of each month. Juveniles were intractable during the summer collection period and thus were not sampled from July-September 2020. A total of 41 fecal samples from adult female, subadult female, and juvenile female animals were collected by zoo staff over both sampling seasons and stored in Whirl-Pak® bags (Nasco, Fort Atkinson, WI, USA) for immediate freezing at -80 °C.

We extracted Microbial DNA from the feces using the PowerFecal Pro DNA kit (QIAGEN, Germantown, MD, USA) after two weeks of storage. We followed manufacturer recommendations, with one modification: after we placed samples in the provided PowerBead Pro tubes (QIAGEN, Germantown, MD, USA) and briefly vortexed, we subjected them to a bead beating step at 4 m/s for 4 min (two cycles of 2 min shaking, with a 1 min pause after each cycle) using a FastPrep-24 bead beater (MP Biomedicals, Santa Ana, CA, USA); this bead beating speed was previously validated for use in DNA extraction for 16S rRNA V3-V4 region sequencing [36]. We eluted extracted DNA in 100 µl of the elution buffer (10 mM Tris) and stored the DNA in elution tubes at -20 °C until the end of the sampling season.

### Sequencing

The Genomic Sciences Laboratory (GSL) at North Carolina State University sequenced the extracted DNA using established methods for the hypervariable V3 and V4 regions of the 16S rRNA gene [37]. The GSL obtained primer pairs from Integrated DNA Technologies (IDT; Coralville, IA, USA) and used them to amplify a sequence approximately 460 base pairs (bp) in length [38]; the exact primer pairs are listed below:

Primers 341F (TCGTCGGCAGCGTCAGATGTGTATAAGAGACAGCCTACGGGNGGCWGCAG) and 805R (GTCTCGTGGGCTCGGAGATGTGTATAAGAGACAGGACTACHVGGGTATCTAATCC).

The GSL performed a limited cycle polymerase chain reaction and added Illumina (San Diego, CA, USA) sequencing adapters and dual-index barcodes to the amplicon target. They then sequenced the V3-V4 region on the Illumina MiSeq platform using paired 300-bp reads and MiSeq v3 reagents.

We imported the resulting raw FastQ files to the CLC Genomics Workbench v21.0.4 with Microbial Genomics Module plugin (QIAGEN, Germantown, MD, USA) and joined forward and reverse reads via the CLC default Illumina platform parameters. We trimmed reads with a 0.05 quality limit and an ambiguous limit of 2 and set read length thresholds between 15 − 1,000 nucleotides. We used the SILVA 16S reference database (v132) (https://www.arb-silva.de) to define operational taxonomic units (OTUs) based on a 97% taxonomic similarity cutoff. We generated an OTU abundance table reformatted it for downstream analysis in RStudio (v1.3.1073) (Boston, MA, USA).

### Bioinformatics and statistical analysis

All statistical analyses were performed using R (version 4.0.2) and RStudio (v1.3.1073). A total of 2,704,600 16S rRNA sequence reads were obtained from 41 samples, with an average of 65,966 ± 4,157 (mean ± SEM) reads per sample (coverage range: 16,005-113,853 reads). Chimeric reads which align to two or more reference sequences were identified and filtered out. A total of 2,982 OTUs were identified across all samples. These 41 samples were rarified to 36,100 reads (with the loss of one sample total) and used to calculate total population and individual results. Rarefaction of the dataset to a set read threshold introduces variable p-value results due to random subsampling. Thus, 50 seed values (reproducible rarefaction permutations) were set, and the resulting histogram of the 50 produced p-values was evaluated to validate significance for each statistical comparison. The 40 data points remaining after rarefaction were filtered twice for age and seasonality statistical comparisons. Age comparisons included 22 data points constituting samples collected from adult, subadult, and juvenile animals during the January-March 2021 collection period (as juvenile animals were not sampled during the July-September 2020 collection period). Seasonality comparisons included 34 samples, constituting all frozen samples from adult and subadult females but excluding juveniles; these samples were used to analyze seasonal effects across all 6 months of the study period. A summary table (Table S1) of the datapoints used for each statistical comparison is available in Additional File 1.

The OTU abundance table was used to calculate taxonomic relative abundance as well as alpha and beta diversity indices. Alpha diversity indices include measures of richness (number of species present) and Shannon diversity (which incorporates both richness and evenness, i.e. relative abundance of taxa). The Shannon diversity index takes into account rare species, making it very sensitive to small changes in diversity. Kruskal-Wallis tests and pairwise Wilcoxon tests were used to assess significant differences in microbial alpha diversity across individuals, age classes, and sample seasons.

Beta diversity indices measure the similarity or dissimilarity between communities [39]. To analyze microbial beta diversity, the relative abundance of each OTU was standardized using the Hellinger transformation, then Bray-Curtis dissimilarity was calculated to create distance matrices. Bray-Curtis dissimilarity values quantify compositional dissimilarity between two sites or groupings based on presence/absence and relative abundance of community membership. Possible values range between 0 and 1, with 0 signifying no bacterial species dissimilarity between two sites and 1 signifying complete species dissimilarity between two sites. Eigenvectors and eigenvalues were calculated from the distance matrices to create nonmetric multidimensional scaling (NMDS) plots, and PERMANOVA analysis was utilized to assess differences in community composition. P-values were adjusted using a False Discovery Rate (FDR) correction. Linear discrimination analysis effect size (LEfSe; https://huttenhower.sph.harvard.edu/galaxy/) was used to identify any OTUs that were significantly enriched per category (i.e., individual, age, reproductive status, or sampling month). Taxa with a linear discriminant analysis (LDA) (log 10) score ≥ ± 3 (hypothesized to be the lower limit for biological relevance; [40, 41]) were reported. It is important to note that while LEfSe provides comparisons of abundance of taxa to the species level, the majority of those reported microbial species are still ambiguous or unclassified. The sequencing of the hypervariable V3 and V4 regions of the 16 S rRNA gene, while valid, can also result in reduced taxonomic accuracy at a species level when compared to sequencing of the entire 16 S gene.

## Results

### Taxonomic relative abundance

The overall microbiomes of juvenile, subadult, and adult female southern white rhinoceros at the North Carolina Zoo were dominated by the bacterial phylum *Firmicutes* (average relative abundance 54%), followed by *Bacteroidetes* (21%), *Spirochetes* (10%), *Fibrobacteres* (8%), *Kiritimatiellaeota* (2%), and *Lentisphaerae* (1%). Phylum and genus level bar charts showed similarities in taxonomic abundance across the majority of individuals, though the abundance of *Firmicutes* and *Fibrobacteres* varied slightly among individuals (Fig. S1).

### Alpha diversity

**Fig. 1:**
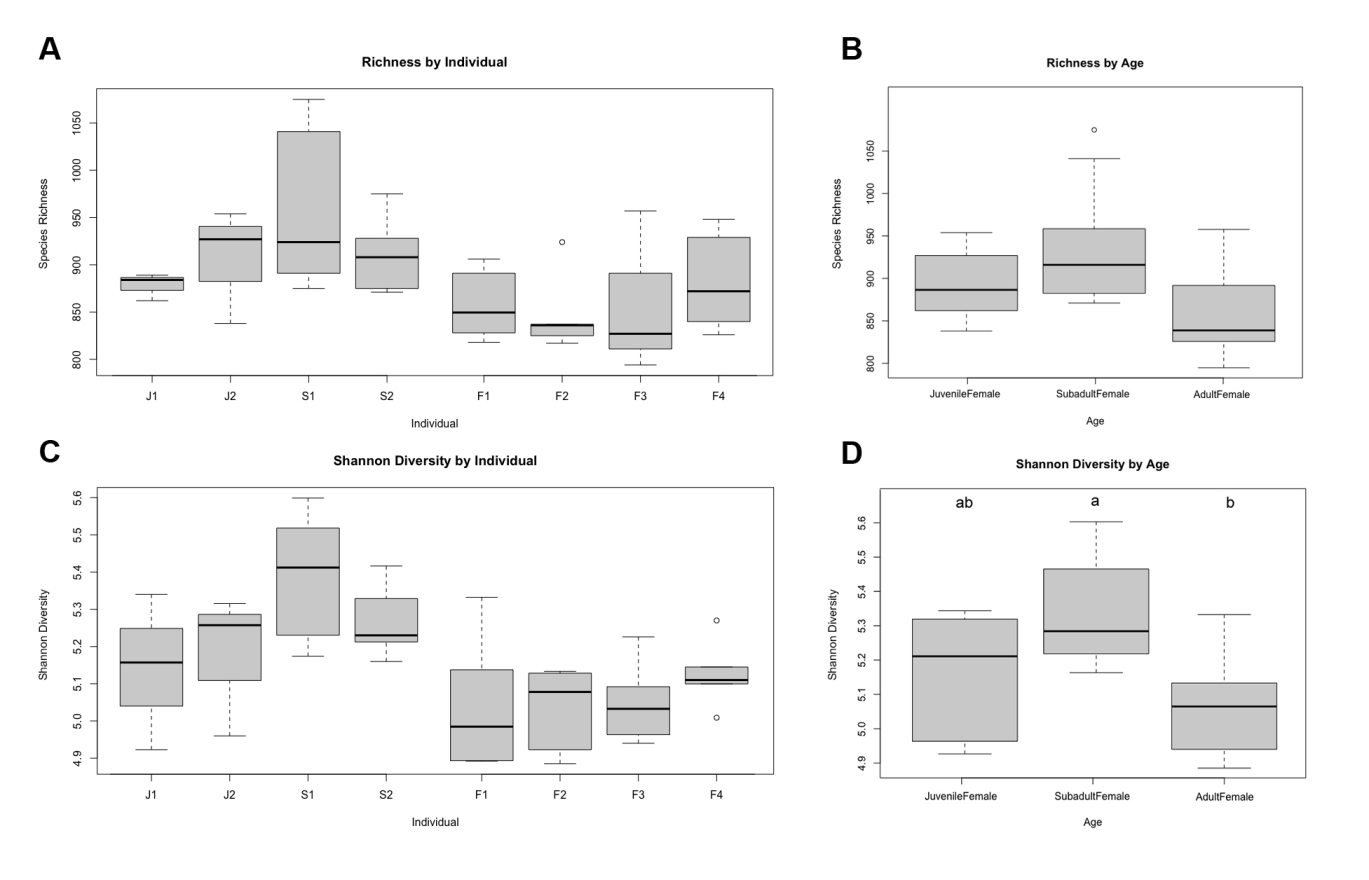
Boxplots comparing alpha diversity as measured by species richness across (**A**) individuals and (**B**) age classes and Shannon diversity across (**C**) individuals and (**D**) age classes for n = 8 female southern white rhinoceros (*Ceratotherium simum simum*) at the North Carolina Zoo in 2020–2021. (**A**) and (**C**) include samples from both seasons, while (**B**) and (**D**) include only samples collected during winter. Error bars represent SEM. Different letters indicate significant differences between age classes (p < 0.05)

**Fig. 2:**
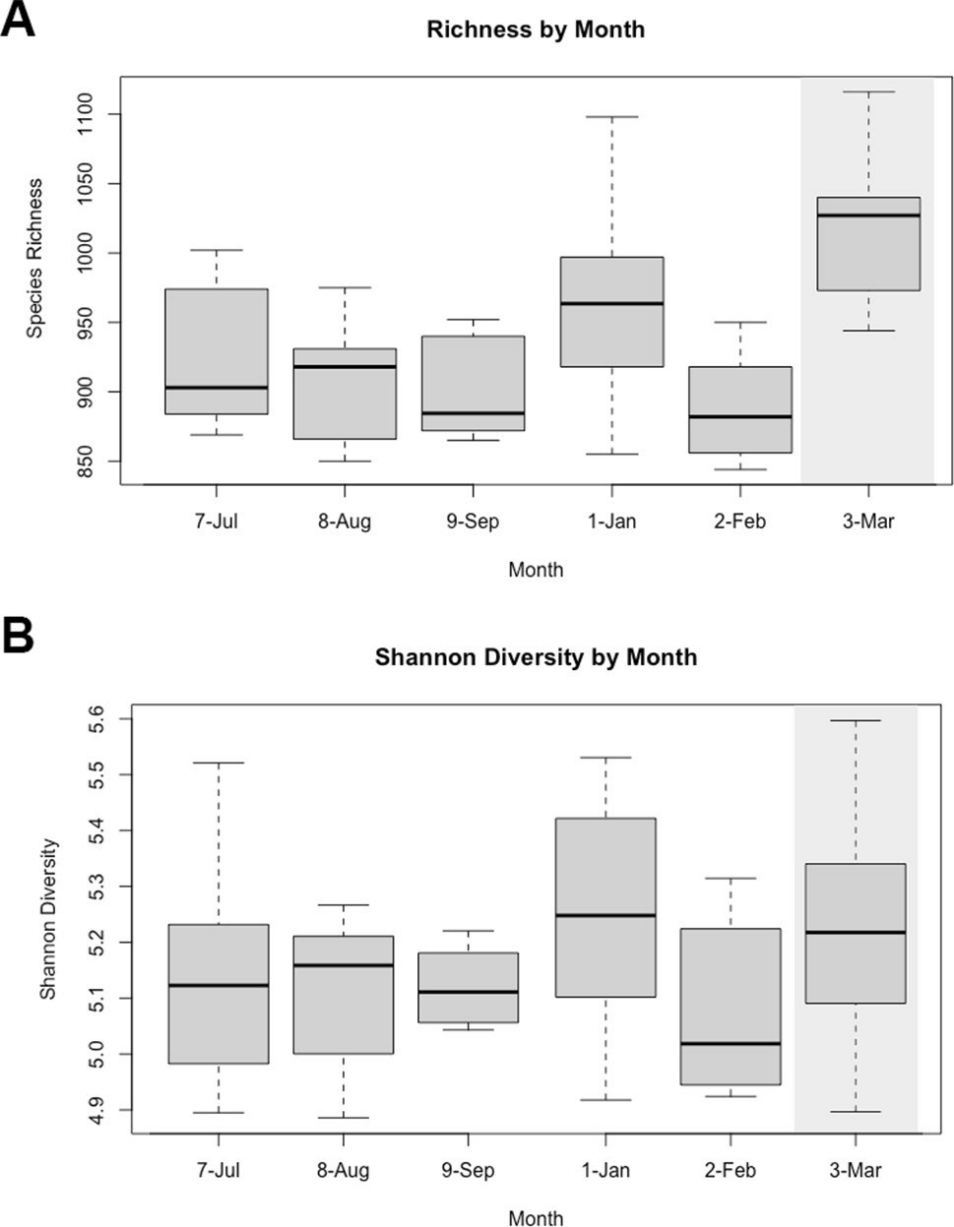
Boxplots comparing (**A**) species richness and (**B**) Shannon diversity of the microbiomes of n = 4 adult female and n = 2 subadult female southern white rhinoceros (*Ceratotherium simum simum*) at the North Carolina Zoo across six months between August of 2020 and March of 2021. Grey background denotes when animals had regular outdoor habitat access (i.e. >20 days per month). Error bars represent SEM

We detected significant differences in species richness and Shannon diversity indices among individuals, age classes, and month of sampling. Kruskal-Wallis *H* tests revealed a significant difference in Shannon diversity (p = 0.01) among individuals, but no difference in richness. Pairwise comparisons using the Wilcoxon rank sum test revealed no significant differences between individuals for any measure of alpha diversity (Fig. 1A C). Kruskal-Wallis *H* tests also revealed significant differences in Shannon diversity (p = 0.008) among age classes during the winter collection period (January-March 2021), likely driven by a significant difference between adult females and subadult females (Wilcoxon rank sum test, p = 0.005) (Fig. 1D). We detected significant differences in species richness (Kruskal-Wallis, p = 0.047) across months, but no differences in Shannon diversity. A Wilcoxon test revealed no significant pairwise differences among months for any measure of alpha diversity when adult and subadult females were compared (Fig. 2).

### Beta diversity

Nonmetric multidimensional scaling (NMDS) plots comparing Bray-Curtis dissimilarity across all samples revealed substantial overlap among individuals, with similar clustering patterns within each age class (Fig. S2). Pairwise comparisons made using a PERMANOVA test revealed no significant differences among individuals.

**Fig. 3:**
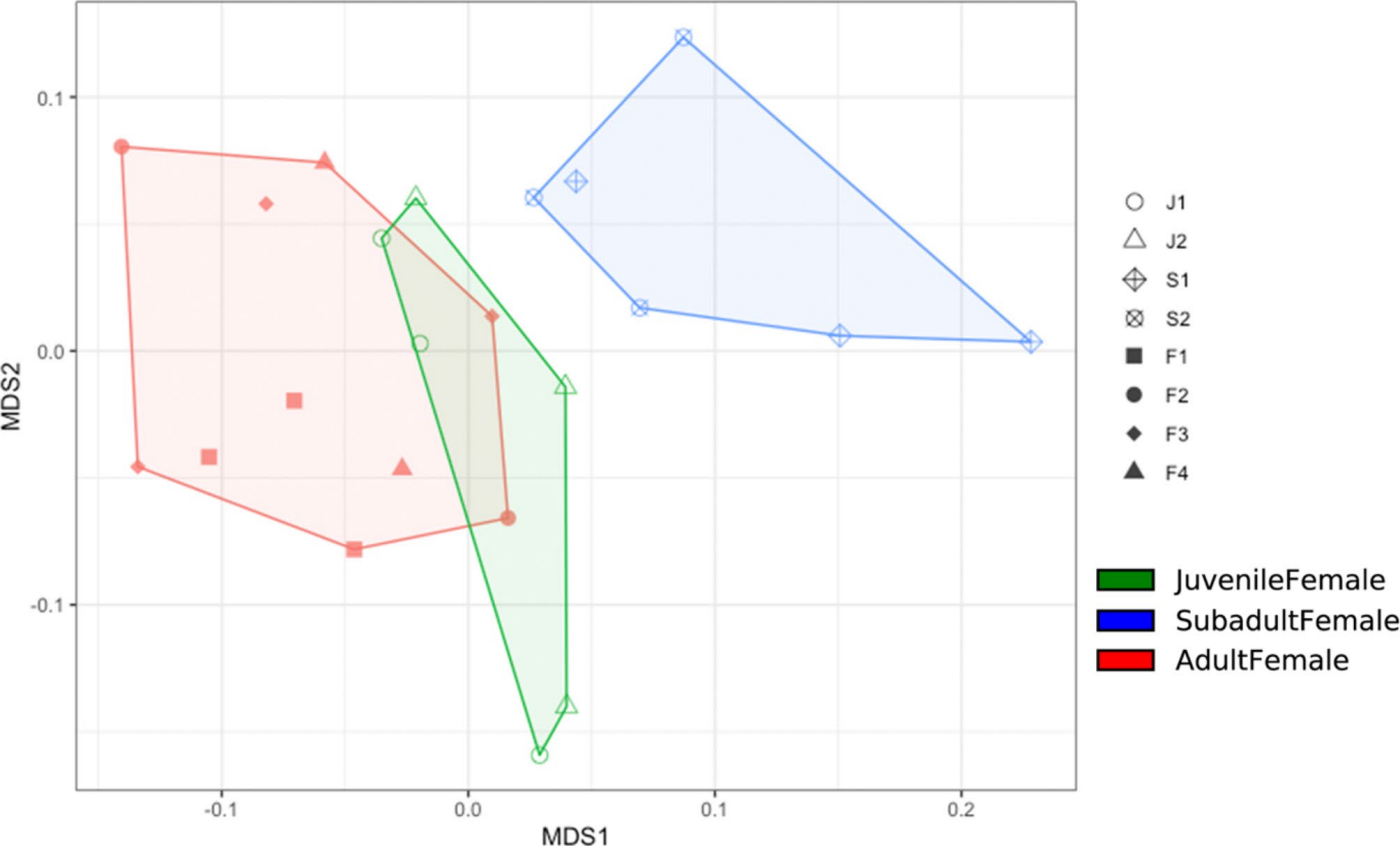
Beta diversity comparisons among age classes as displayed by multidimensional scaling (MDS) plot for n = 8 female southern white rhinoceros (*Ceratotherium simum simum*) sampled at the North Carolina Zoo from January-March 2021

**Fig. 4:**
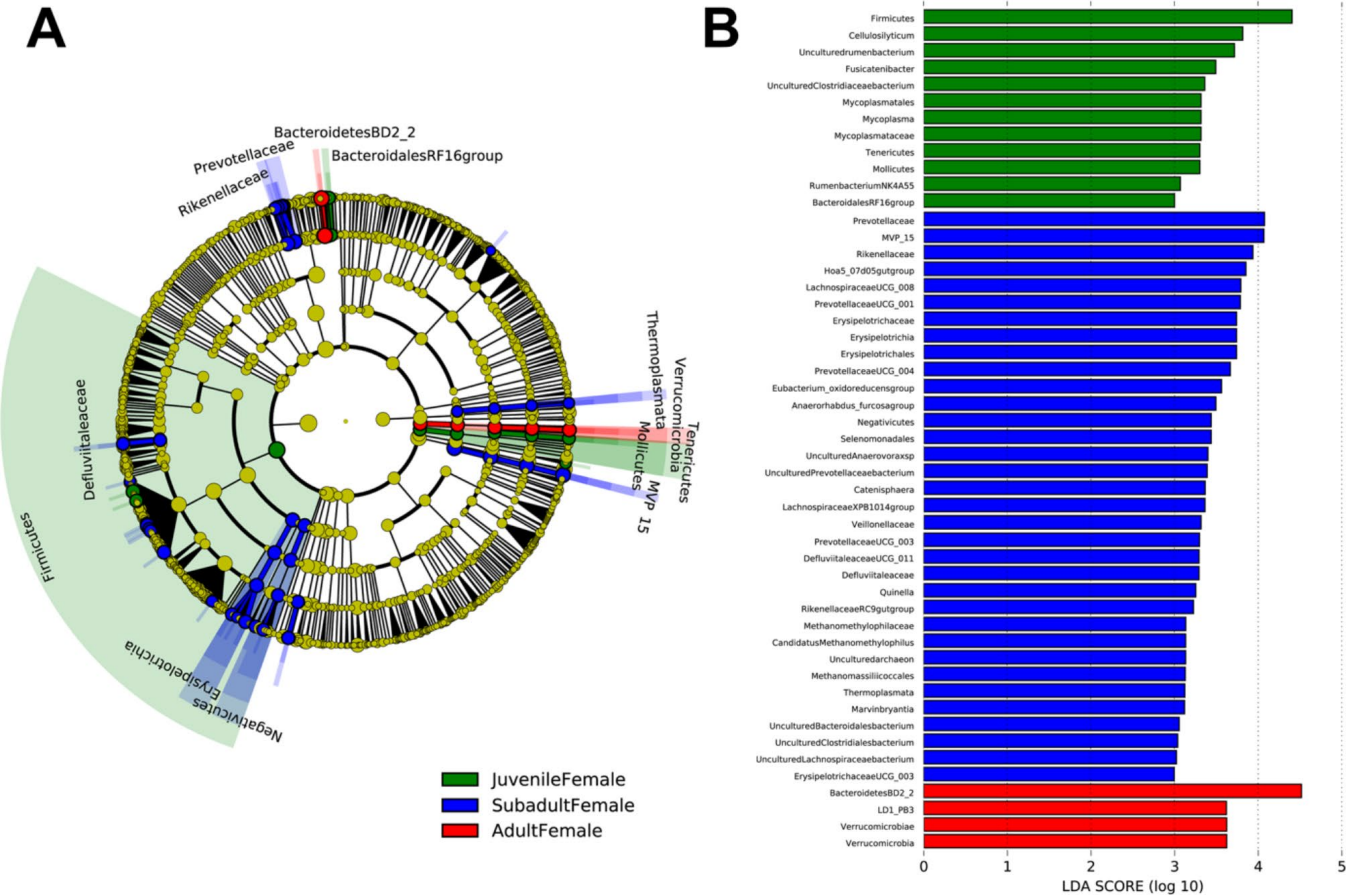
(**A**) Linear discriminant analysis effect Size (LEfSe) cladogram and (**B**) linear discriminant analysis (LDA) plot comparing differentially enriched taxa by age class for n = 8 female southern white rhinoceros (*Ceratotherium simum simum*) sampled at the North Carolina Zoo from January-March 2021

An NMDS plot comparing the three age classes sampled in the winter collection period (January-March 2021) showed that adult and juvenile microbial community clusters overlapped slightly and were both distinct from the subadult cluster (Fig. 3). A PERMANOVA analysis indicated that every age group was significantly different (p < 0.05) in community composition (Table 3). LEfSe analysis revealed significantly enriched taxa distinct to each of the three age classes (Fig. 4A and B). Adult female rhinoceros harbored the fewest enriched taxa of the age groups, comprising members of the phylum *Verrucomicrobia* (specifically uncultured taxa within the order *LD1-PB3* (p = 0.004)) and the family *Bacteroidetes BD2-2* (p = 0.05). In contrast, subadult females possessed the greatest number of differentially enriched taxa (68), compared to 20 taxa in juveniles and 9 taxa in adults (Additional file 2). Specifically, subadults hosted several taxa within the *Bacteroidetes* phylum, including the family *Prevotellaceae* (p = 0.001, driven by the genera *Prevotellaceae* UCG-001 (p = 0.001), UCG-003 (p = 0.023), and UCG-004 (p = 0.004)) and *Rikenellaceae* (p = 0.004, driven by the *Hoa5-07d05* gut group (p = 0.008) and *RC9* gut group (p = 0.001) genera). Members of the *Firmicutes* families *Defluviitaleaceae* (p = 0.016) and *Family XIII* (p = 0.002) were also enriched in subadults, as well as the genera *Eubacterium oxidoreducens* group (p = 0.01), *Acetitomaculum* (p = 0.014), *Lachnospiraceae* UCG-006 (p = 0.011), *Lachnospiraceae* UCG-008 (p = 0.013), *Lachnospiraceae XPB1014* group (p = 0.049), *Marvinbryantia* (p = 0.047), *Ruminococcus* 1 (p = 0.046), and the species *Eubacterium ramulus* (p = 0.021). Several members of the *Firmicutes* class *Erysipelotrichia* (p = 0.001) were enriched, driven by species within the *Anaerorhabdus furcosa* group (p = 0.002), *Catenisphaera* (p = 0.005), and *Erysipelotrichaceae* UCG-003 (p = 0.002*)* genera. Similar enrichment in the *Firmicutes* class *Negativicutes* (p = 0.013) was revealed, driven by members of the *Selenomonadales* families, *Acidaminococcaceae* (p = 0.044) and *Veillonellaceae* (p = 0.035). Within *Veillonellaceae*, *Quinellla* species were significantly and distinctly enriched (p = 0.006). Several ambiguous taxa were also enriched, including those within the *Kiritimatiellaeota* order *WCHB1-41* (p = 0.038), *Spirochaetes* class *MVP-15* (p = 0.047), *Planctomycetes* family *Pirellulaceae* (p = 0.015), and *Actinobacteria* family *Microbacteriaceae* (p = 0.03). In addition, members of the class *Thermoplasmata* were enriched, driven by taxa within the order *Methanomassiliicoccales* (p = 0.018) and genus *Candidatus Methanomethylophilus* (p = 0.022).

Juvenile females harbored fewer enriched taxa, which were mostly constrained to the *Firmicutes* and *Tenericutes* phyla. Enrichment in *Firmicutes* was driven by members of the *Lachnospiraceae* genus *Fusicatenibacter* (p = 0.009), an uncultured *Clostridiaceae* bacterium within the *Papillibacter* genus (p = 0.045), two uncultured members of *Ruminiclostridium 9* (p < 0.05), and *Rumenbacterium NK4A55* of the *Anaerovibrio* genus (p = 0.004). Enrichment in *Tenericutes* was driven by an uncultured member of the *Mollicutes* order *Mycoplasmatales* (p = 0.019). As previously mentioned, juveniles were sampled for only one season and the dataset is thus less complete than that of subadults and adults.

**Table 3:** PERMANOVA statistical comparisons of Bray-Curtis dissimilarity in microbial community composition across three age classes of n = 8 female southern white rhinoceros (*Ceratotherium simum simum*) housed at the North Carolina Zoo from January-March 2021

	Adult Female	Juvenile Female
**Juvenile Female**	0.005**	-
**Subadult Female**	0.003**	0.023**

Significance codes: ‘**’ 0.01

**Fig. 5:**
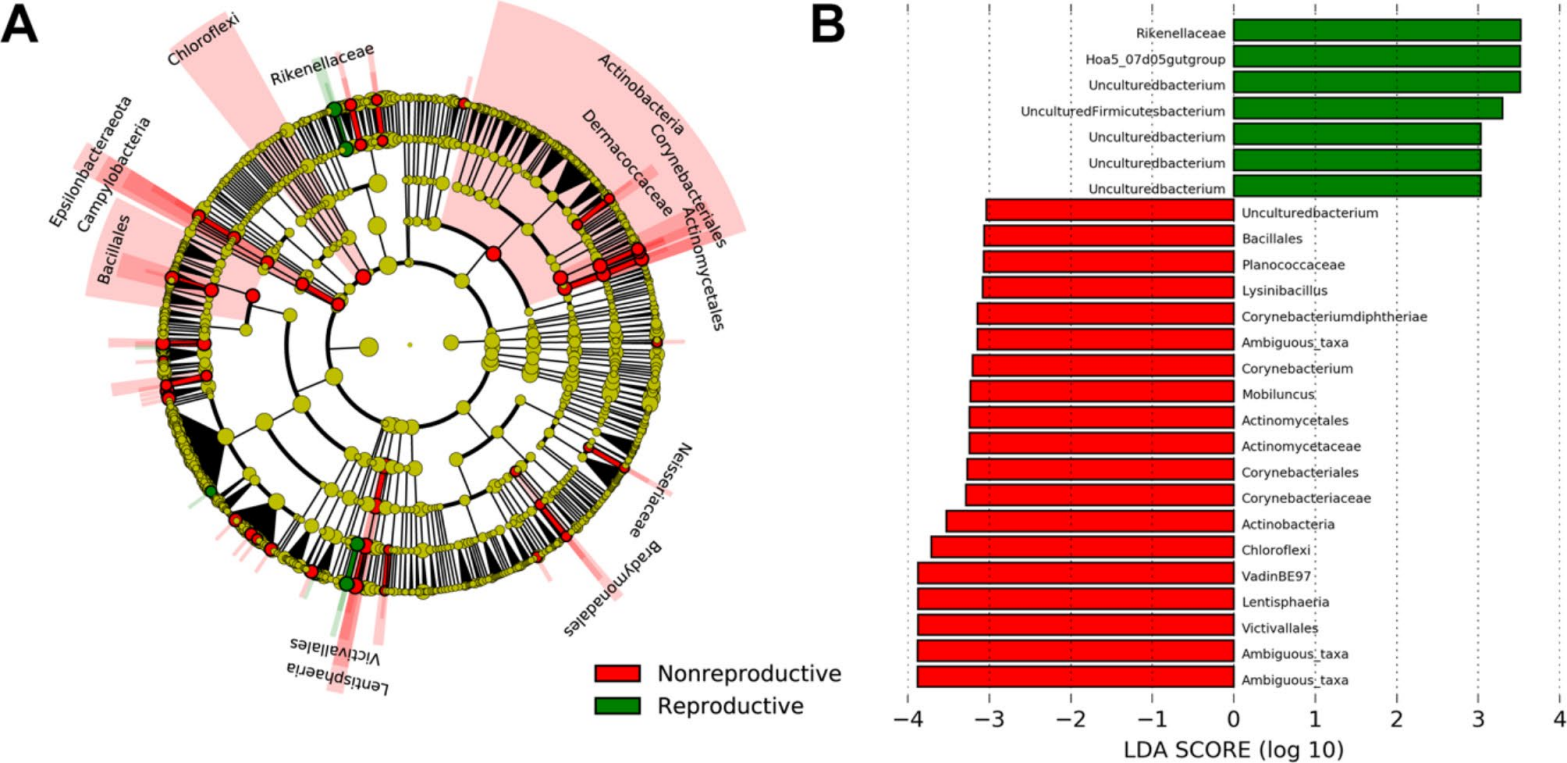
(**A**) Linear discriminant analysis effect Size (LEfSe) cladogram and (**B**) linear discriminant analysis (LDA) plot comparing differentially enriched taxa for n = 2 reproductive and n = 2 nonreproductive female southern white rhinoceros (*Ceratotherium simum simum*) sampled at the North Carolina Zoo between 2020–2021

A third LEfSe analysis revealed several differentially enriched taxa between reproductively active (n = 2) and nonreproductive (n = 2) adult female rhinoceros (Fig. 5). Reproductively active females were enriched in members of the *Bacteroidetes* family *Rikenellaceae* (p = 0.027), driven by an uncultured species of the *Hoa5-07d05* gut group (p = 0.036), as well as uncultured taxa within the *Firmicutes* family *Lachnospiraceae* (p = 0.025) and *Kiritimatiellaeota* order *WCHB1-41* (p = 0.031). Nonreproductive females were enriched in the *Actinobacteria* phylum (p = 0.01), driven by the orders *Actinomycetales* (p = 0.007) (specifically members of the family *Actinomycetaceae* (p = 0.007) and the *Mobiluncus* genus (p = 0.001)) and *Corynebacteriales* (p = 0.007) (driven by *Corynebacterium diphtheriae* (p = 0.004)). Nonreproductive females also harbored enriched phyla *Chloroflexi* (p = 0.036) and *Lentisphaerae* (p = 0.006, driven by uncultured members of the *Victivallales* family *VadinBE97* (p = 0.006)); and *Firmicutes* order *Bacillales* (p = 0.05), driven by members of the genus *Lysinibacillus* (p = 0.028) within the family *Planococcaceae* (p = 0.04)).

**Fig. 6:**
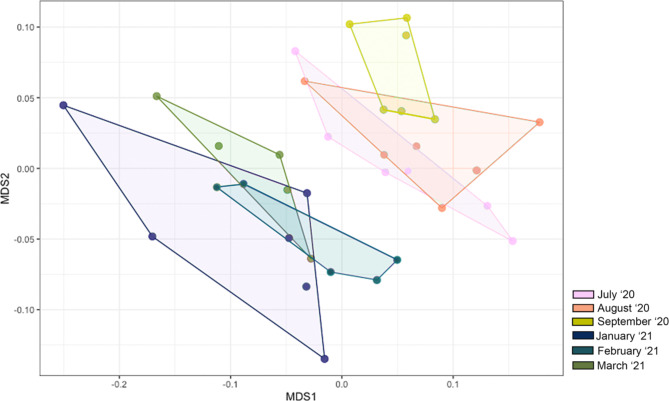
Beta diversity comparisons among six sampling months as displayed by multidimensional scaling (MDS) plot for n = 2 subadult female and n = 4 adult female southern white rhinoceros (*Ceratotherium simum simum*) sampled at the North Carolina Zoo from 2020–2021

An NMDS plot comparing community composition for the n = 4 adult and n = 2 subadult females across six months revealed a distinct trend in clustering based on seasonality (Fig. 6). The winter sample collection months of January, February, and March shared overlap in clustering, and the clusters were discrete from the summer sample collection months of July, August, and September. The summer months also clustered together, with the majority of overlap between July and August. A PERMANOVA analysis showed that none of the winter months differed significantly. Summer months all differed from winter months (p < 0.05), and there was no significant difference between July and August. However, September was significantly different from every other month (p < 0.01; Table 4).

**Fig. 7:**
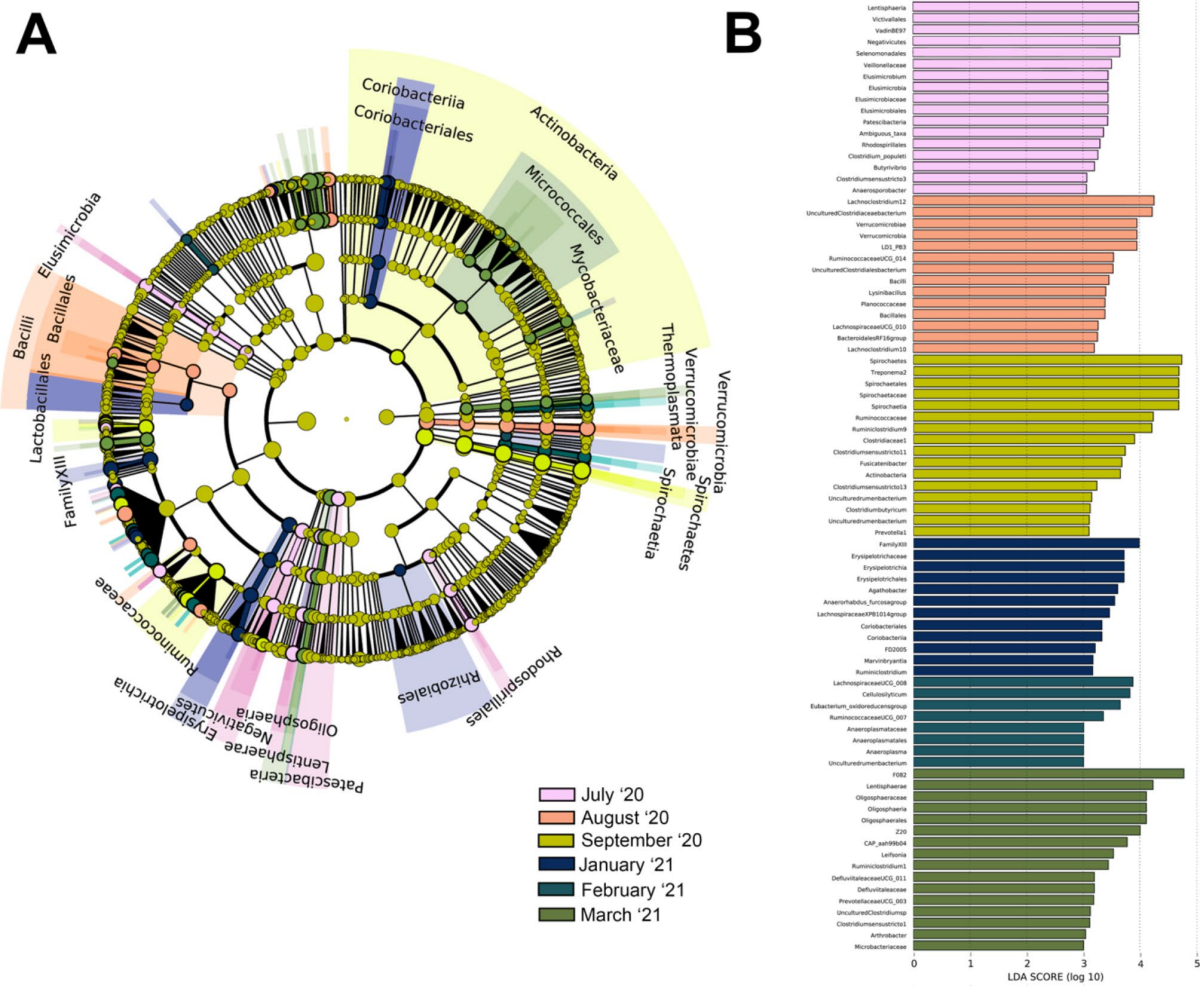
(**A**) Linear discriminant analysis effect Size (LEfSe) cladogram and (**B**) linear discriminant analysis (LDA) plot comparing differentially enriched taxa for n = 2 subadult female and n = 4 adult female southern white rhinoceros (*Ceratotherium simum simum*) sampled at the North Carolina Zoo from 2020–2021

LEfSe analysis revealed differentially abundant taxa distinct to each month, with March having the most numerous enriched taxa (n = 44), followed by January (n = 41) (Fig. 7A and B). Only those taxa which were classified are listed below; a spreadsheet of all LDA scores and p-values for each month is available in Additional file 2.

Of the summer collection months (July-September 2021), July had the highest number of enriched taxa (n = 32) including *Elusimicrobium* (p < 0.001), *Patescibacteria* (p = 0.001), *Rhodospirillales* (p = 0.036), *Butyrivibrio* (p = 0.031), *Anaerosporobacter* (p < 0.001), *Lentisphaerota* (p = 0.034, driven by *Victivallales* (p = 0.034)), and *Negativicutes* (p = 0.018, driven by *Veillonellaceae* (p = 0.021)). August was enriched in *Lachnoclostridium* 10 (p = 0.008) and 12 (p < 0.001), *Verrucomicrobia* (p = 0.002, driven by *LD1 PB3* (p = 0.002)), *Ruminococcaceae* UCG-014 (p = 0.024), *Bacillales* (p = 0.01, driven by *Lysinibacillus* (p = 0.03)), and *Bacteroidales RF16* group (p = 0.003). September was enriched in *Actinobacteria* (p = 0.032), *Spirochaetes* (p = 0.02, driven by *Treponema* (p = 0.018)), *Ruminococcaceae* (p = 0.041), *Ruminiclostridium* 9 (p = 0.001), *Prevotella* 1 (p = 0.028), and *Fusicatenibacter* (p = 0.009). In addition, several *Clostridiaceae* (p = 0.003) genera and species were enriched including *Clostridium sensu stricto* 11 (p < 0.001), *Clostridium sensu stricto* 13 (p = 0.008), and *Clostridium butyricum* (p = 0.02).

Of the winter months, January was enriched in *Coriobacteriales* (p = 0.01), *Agathobacter* (p = 0.005), *Marvinbryantia* (p = 0.014), *Ruminiclostridium* (p = 0.017), *Clostridiales Family XIII* (p < 0.001), *Lachnospiraceae* genus *FD2005* (p < 0.001), *Erysipelotrichia* (p < 0.001, driven by *Anaerorhabdus furcosa* group (p < 0.001)), and an uncultured *Cyanobacteria* rumen bacterium of the order *Gastranaerophilales* (p = 0.041). February was enriched in *Eubacterium oxidoreducens* group (p = 0.01), *Cellulosilyticum* (p = 0.025), *Lachnospiraceae* UCG-008 (p = 0.03), *Anaeroplasma* (p = 0.006), and *Ruminococcaceae* UCG-007 (p = 0.015). March had the highest number of enriched taxa across all months (n = 44), including *Lentisphaerae* (p = 0.041, driven by uncultured member of *Oligosphaeraceae* genus *Z20* (p < 0.001)), *Leifsonia* (p = 0.027), Defluviitaleaceae (p = 0.009), *Ruminiclostridium* 1 (p < 0.001), *Prevotellaceae* UCG-003 (p = 0.023), *Arthrobacter* (p = 0.002), *Microbacteriaceae* (p = 0.047), *Bacteroidales* families *F082* (p = 0.007) and *CAP-aah99b04* (p = 0.02), and *Clostridium sensu stricto* 1 (p = 0.024). The daily average temperature in Asheboro, NC for March 2021 was ~ 12 °C, making March the first of the winter months to facilitate the regular (i.e. >20 days per month) rotation of both the rhinoceros groups onto the Grasslands habitat.

**Table 4:** PERMANOVA statistical comparisons of Bray-Curtis dissimilarity in microbial community composition across 6 months. Samples collected from n = 2 subadult and n = 4 adult female southern white rhinoceros (*Ceratotherium simum simum*) housed at the North Carolina Zoo between 2020–2021

	January	February	March	July	August
**February**	0.094	-	-	-	-
**March**	0.068	0.094	-	-	-
**July**	0.005**	0.016*	0.005**	-	-
**August**	0.005**	0.005**	0.005**	0.126	-
**September**	0.005**	0.005**	0.005**	0.013*	0.005**

Significance codes: ‘**’ 0.01 ‘*’ 0.05

## Discussion

### Total population taxonomic abundance

Here we have characterized the gut microbiomes of eight female southern white rhinoceros at the North Carolina Zoo, a managed, reproductively active population valuable to the preservation of the species. The dominance of *Firmicutes* and *Bacteroidetes* within this population coincides with a previously published overview of Rhinocerotidae gut microbial relative abundances. Across southern white rhinoceros, black rhinoceros (*Diceros bicornis*), Sumatran rhinoceros (*Dicerorhinus sumatrensis*) and greater one-horned rhinoceros (*Rhinoceros unicornis*), the two most dominant phyla were indeed *Firmicutes* (range; 66.3–51.0%) and *Bacteroidetes* (39.8–23.4%) [32]. *Verrucomicrobia* (7.6–1.9%), *Spirochetes* (3.1–1.1%), *Actinobacteria* (1.04–0.03%), and *Fibrobacteres* (2.14–0.19%) were also dominant across the four rhinoceros species [32], though their ranked abundances do not coincide with the current study: the phylum *Actinobacteria* was not present above 1% relative abundance in the majority of the North Carolina Zoo individuals, and *Verrucomicrobia* was only found at > 1% abundance in one individual (F4 in September 2020). Differences among separate populations of managed southern white rhinoceros may be attributed to a variety of factors, including facility-specific diet (e.g. locally-sourced hay), geographic location, and the characteristics described here such as age, seasonality of sampling, and reproductive status.

A previous study by McKenzie et al. has shown that Rhinocerotidae are one of the few families to experience increased microbial diversity under management compared to their wild conspecifics [11]. This is interesting, as many managed animal species have been noted to experience a decrease in microbial richness and diversity when compared to wild populations [11, 42–44]. This decrease has been attributed to limited environmental exposure, the usage of antibiotics and other medical interventions, lack of interaction with other species, and lack of dietary diversity compared to wild conditions, among other factors. However, the McKenzie et al. study was limited by small sample size (n = 6 southern white rhinoceros, 3 managed and 3 wild; n = 7 black rhinoceros, 6 managed and 1 wild) and unknown wild individual characteristics (i.e. age, sex, health status) [11]. Most comparisons of managed vs. wild animal gut microbiomes are similarly constrained, making valid inferences challenging and further emphasizing the need for additional studies to confirm the effects of management on gut microbial diversity.

### Age-related gut microbiome differences

We identified age-related changes in gut microbial diversity and composition in the female southern white rhinoceros gut microbiome (Figs. 1D and 3; Table 3), similar to several previous studies and host systems, including humans and horses [45–47]. Notably, Bray Curtis distance (i.e., inter-individual variation) within age class increases with age from juvenile to subadult to adult, in contrast with earlier studies of gut microbial succession across development [48]. It is important to note that the microbial community compositions of juveniles and adults are less dissimilar than either juveniles and subadults or subadults and adults. One might expect the gut microbial community compositions of cow-calf pairs to cluster closely together, as mammals likely receive their first vertical microbial inoculation during parturition [49–51]. Juveniles continue to establish their gut microbiomes horizontally via environmental and social contact and mediate microbial species abundance via the consumption of milk and feed [50, 52]. While the community clusters of the two juvenile individuals did share some overlap with the adult female rhinoceros, there was no specific overlap with their mothers’ clusters (Fig. 3). We propose that the dynamics within this population might result from the relatively greater dietary diversity consumed by adult and subadult females compared to juveniles. Juvenile microbiomes are also likely in a transitory state during weaning, after having been colonized mainly by milk metabolizing microbiota. Both juveniles in this study were approaching the average weaning age (~ 1.5 years) for female southern white rhinoceros calves [53, 54].

Juvenile animals were differentially enriched in several beneficial taxa associated with glucose and fiber fermentation in the guts of hindgut fermenters and ruminants, including *Fusicatenibacter*, *Papillibacter*, *Rumenbacterium NK4A55*, *Ruminiclostridium* 9, and *Pyramidobacter*. Conversely, they were enriched in *Mycoplasma* sp. which are considered pathogenic bacteria and have been associated with several deleterious conditions in horses, including endometritis, vulvitis, and infertility [55]. However, juveniles in this population were considered healthy and asymptomatic for *Mycoplasma* infection.

Significant differences (p < 0.01) in subadult female and adult female animal gut microbiome alpha and beta diversity were present (Fig. 1D; Table 3) though similarities in lifestyle make inferences challenging; both age classes overlapped in housing, outdoor access, and diet. The two subadult females were potentially beginning puberty as they were around three years of age, so hormonal shifts may have affected their microbial diversity [5, 56]. Studies into human and murine models have suggested that the sex hormone estrogen has bidirectional interactions with gut microbiota, and a previous study into southern white rhinoceros dietary estrogenicity proposed similar interactions [7, 57]. However, the rhinoceros study consisted only of adult individuals, with no investigation into age-related differences in estrogenicity and gut microbiome interaction [7]. Subadult animals were enriched in *Rikenellaceae*, *Prevotellaceae*, *Quinella*, and *Marvinbryantia* (Fig. 2). *Rikenellaceae* and *Prevotellaceae* have been proposed to contribute to fiber degradation and metabolite production in southern white rhinoceros [7]. *Quinella* is also responsible for digestion as a large propionate-producing rumen bacterium, and *Marvinbryantia* has been similarly implicated in microbial fermentation processes [58–60]. These beneficial bacteria may be enriched in the subadult rhinoceros gut as the microbiome continues to progress toward the more stable adult climax composition.

### Reproductive status and the gut microbiome

There were several distinct taxa differentially enriched in adult female rhinoceros based on reproductive status; reproductive females (n = 2) have both had at least two offspring at the North Carolina Zoo, while nonreproductive females (n = 2) are sexually mature but have not calved since arriving at the zoo in 2007 (though they were exposed to the same bulls for the same amount of time). Interestingly, reproductive females were enriched in *Rikenellaceae* (specifically members of the *Hoa5-07d05* gut group*)*, which is a known saccharolytic family of bacteria that has been hypothesized by Williams et al. [7] to affect white rhinoceros fertility by contributing to the catalyzation of phytoestrogen transformation. Reproductive females were also enriched in fewer taxa than nonreproductive females, suggesting a more stringent immune regulation of their gut microbiome. In contrast, nonreproductive females were enriched in less specialized and less beneficial taxa, such as members of the *Corynebacteriales* family and *Mobiluncus* genus. Interestingly, *Corynebacterium diphtheriae* was specifically enriched in nonreproductive females but had not been previously isolated in rhinoceros and has only rarely been observed in horses and other domestic animals [61–63]. As *C. diphtheriae* exists predominantly in humans, it is probable that the bacterium is transmitted to managed animals, such as zoo rhinoceros, by close human contact [63]. Regardless, the strain is likely nontoxigenic given the asymptomatic status of the nonreproductive females. *Mobiluncus* species, however, are heavily associated with adverse reproductive outcomes and have been connected to inflammation of the uterine lining in bovines [64] and bacterial vaginosis in primates with associated dysbiotic cervicovaginal microbiome [65–68]. *Mobiluncus curtsii* and *Mobiluncus mulieris* specifically have been associated with spontaneous preterm birth in humans [69], with a possible mechanism being the disruption of the cervical epithelial barrier via inflammatory and microRNA mediators [68]. While the presence of *Mobiluncus* sp. within the cervicovaginal microbiome is well-documented, its presence within the gut microbiome and ensuing effect on the host is less understood and we cannot state with any certainty that the unclassified members of *Mobiluncus* sp. enriched in the non-reproductive rhinoceros of this study are detrimental or benign without further research. The cervicovaginal microbiome is partially maintained through translocation of gut microbiota via the rectum, which serves as a reservoir; thus, it is plausible that the unclassified species of *Mobiluncus* identified is a normal member of the fecal microbiome in this population [70–72].

Long-term elevated reproductive hormones associated with pregnancy and lactation may drive differences in gut microbiome between reproductive and nonreproductive southern white rhinoceros females. Research into black rhinoceros found that gut microbial composition is significantly altered during pregnancy and post-parturition, possibly mediated by changes in maternal metabolism and oxytocin production [73]. Antwis et al. [73] also found that *Actinobacteria* decreases in abundance in pregnant black rhinoceros, similarly to this study where *Actinobacteria* are significantly more abundant in nonreproductive female southern white rhinoceros compared to reproductive females (Fig. 5). Further research is necessary to produce direct evidence of gut microbiome affecting reproductive status in southern white rhinoceros, assess the exact mechanisms and certain gut microbiota responsible, and discern why specific individuals are more susceptible to harboring reproductively deleterious microbes.

### Seasonal effects on gut microbiome

Significant differences in species richness and Bray-Curtis dissimilarity across sampling months were identified and seasonal trends were observed (Table 4; Fig. 6). Seasonal dynamics have previously been documented in other hindgut fermenters under management, including gorillas [16] and horses [31]. Horses have been used as the domestic animal model for rhinoceros species due to similarities in gut morphology and hindgut microbial fermentation although differences likely exist [56]. At the start of this study, we hypothesized that feces collected from the population during the summer collection period (July-September 2020) would reflect higher levels of species richness than the winter collection period (January-March 2021) because the rhinoceros would normally have seasonal access to the 16-hectare habitat and all of the gut microbiome influences that come with it (i.e. contact with environmental microbiota, potential interactions with fecal microbiota of other species, and the addition of several grass species to their diet). This expectation was based on historical rhinoceros husbandry routines at the North Carolina Zoo but was affected by a cyanobacterial bloom in the lake of the Grassland habitat during the summer of 2020, resulting in the rhinoceros not having access to the natural grazing throughout the entirety of the summer collection period (July-September). The logic of the hypothesis was sound, however, as rhinoceros were rotated back out onto the Grasslands habitat during the winter collection period (January-March 2021) subject to ambient temperature (> 0 °C for nonreproductive females; >7 °C for cow-calf pairs) and January and March subsequently had the highest median species richness and Shannon diversity of all months (Fig. 2). March 2021 (the first of the winter collection months to facilitate regular outdoor habitat access i.e., > 20 days per month) was also enriched in many taxa associated with aquatic and terrestrial environments which were likely picked up from the soil, decaying plant matter, and freshwater lake of the habitat; these bacteria included members of the families *Defluviitaleaceae* and *Microbacteriaceae* and genera *Leifsonia* and *Arthrobacter*.

### Limitations

This study has several limitations, the first of which is the small total population size (n = 8) sampled, which limits the robustness of statistical inferences. In addition, the population of animals in this study belonged to one facility, thus these results are most relevant to that facility and may not be extrapolated to managed southern white rhinoceros populations as a whole. To illustrate, diet is one of the largest drivers of variation in the gut microbiome, and the diet at this facility would be unique (i.e. locally-sourced hays and native grasses) and unlike that of any facility outside its immediate geographic range. This study sampled fecal microbiota as a proxy for gut microbiota, though recent research has proposed that feces is inadequate at representing the microbiome present in both the contents and mucosa of the gastrointestinal tract [74]. In addition, different parts of the gastrointestinal tract possess different abundances of microbial families which may not be apparent in feces [75]. True sampling of the intestinal tract would involve highly invasive or fatal biopsies that are inappropriate for studies with endangered animals. As half of the rhinoceros in this study were not tractable enough for per rectal sampling, the non-invasive sampling of feces after defecation was necessary. This study used Illumina sequencing of the ~ 460 bp V3 to V4 region of the 16S rRNA gene to identify bacterial OTUs and analyze composition compared to using the full ~ 1500 bp 16 S gene. This compromise produced high throughput results at a lower cost than other sequencing methods but reduced taxonomic accuracy at a species level. Finally, sequencing of samples took place after the completion of each sampling period, meaning that not all samples were sequenced at the same time. While this has the potential to add sequencing variability stemming from different sequencing runs (i.e., “batch effects”) [76], the utilization of the same laboratory, procedure, and technicians limited this variation as much as possible.

## Conclusions

This study has revealed novel age and seasonal-related differences in microbial diversity and community clustering within the female southern white rhinoceros population at the North Carolina Zoo. Further research with managed populations of southern white rhinoceros of varied age groups and sexes would be ideal for validating age-related trends and also allow for the investigation of sex as another possible driver of microbial variation within the species. In addition, differentially enriched taxa were revealed between reproductively active and currently nonreproductive female rhinoceros within the population, with nonreproductive females specifically enriched in microbial taxa belonging to a genus (i.e. *Mobiluncus*) that possesses species associated with poor pregnancy outcomes in other animals when identified in the cervicovaginal microbiome. Though we cannot conclude with any certainty that the abundance of *Mobiluncus* in the gut microbiome of these two nonreproductive rhinoceros is responsible for poor reproductive outcomes, we suggest that *Mobiluncus* sp. are a microbial marker of interest and should be more thoroughly assessed by future studies. Regardless, this reproductively active managed population of rhinoceros provides valuable baseline data for future comparative studies with larger or differently managed populations at other facilities.

## Electronic supplementary material

Below is the link to the electronic supplementary material.

Supplementary Material 1

Supplementary Material 2

## Data Availability

Raw sequence data are available from the NCBI Sequence Read Archive (SRA) database at https://www.ncbi.nlm.nih.gov/sra under the BioProject ID PRJNA859535. Original R script, metadata, and unrarefied OTU table are available in GitHub (https://github.com/Flyredbird/Rhinobome). Raw LEfSe Analysis data is available in Additional File 2.
