# Supplementary material for: Effects of age, seasonality, and reproductive status on the gut microbiome of Southern White Rhinoceros (Ceratotherium simum simum) at the North Carolina zoo
Source: Anim Microbiome. 2023 May 5;5:27. doi: 10.1186/s42523-023-00249-5 (PMC10163733; doi:10.1186/s42523-023-00249-5)
Supplement: Supplementary file 2 — Supplementary Material 2 [file 42523_2023_249_MOESM2_ESM.pdf]

Additional File 1

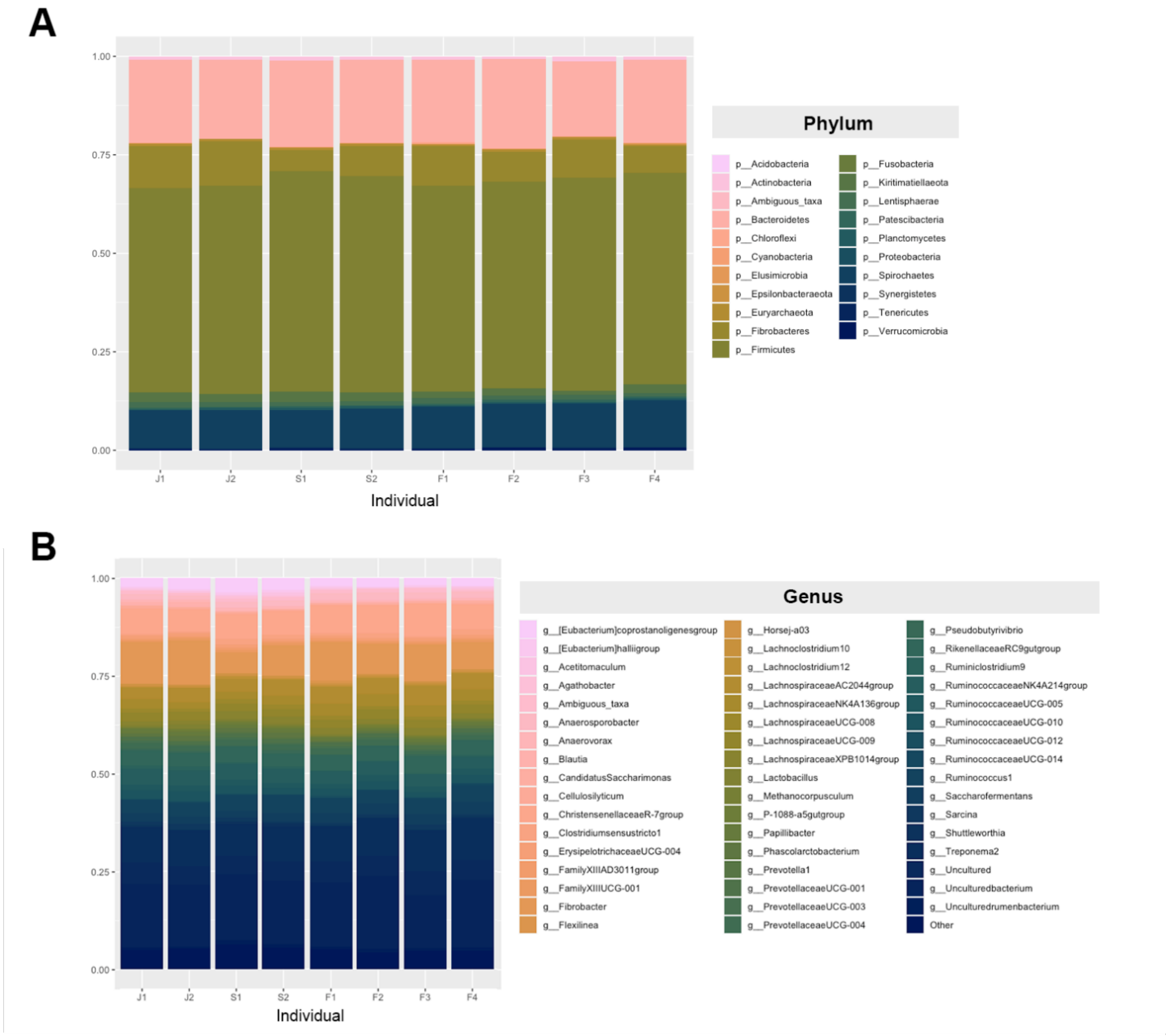

Figure S1. (A) Phylum - and (B) genus - level bar charts representing microbial community composition across n=8 female southern white rhinoceros at the North Carolina Zoo between 2020-2021.

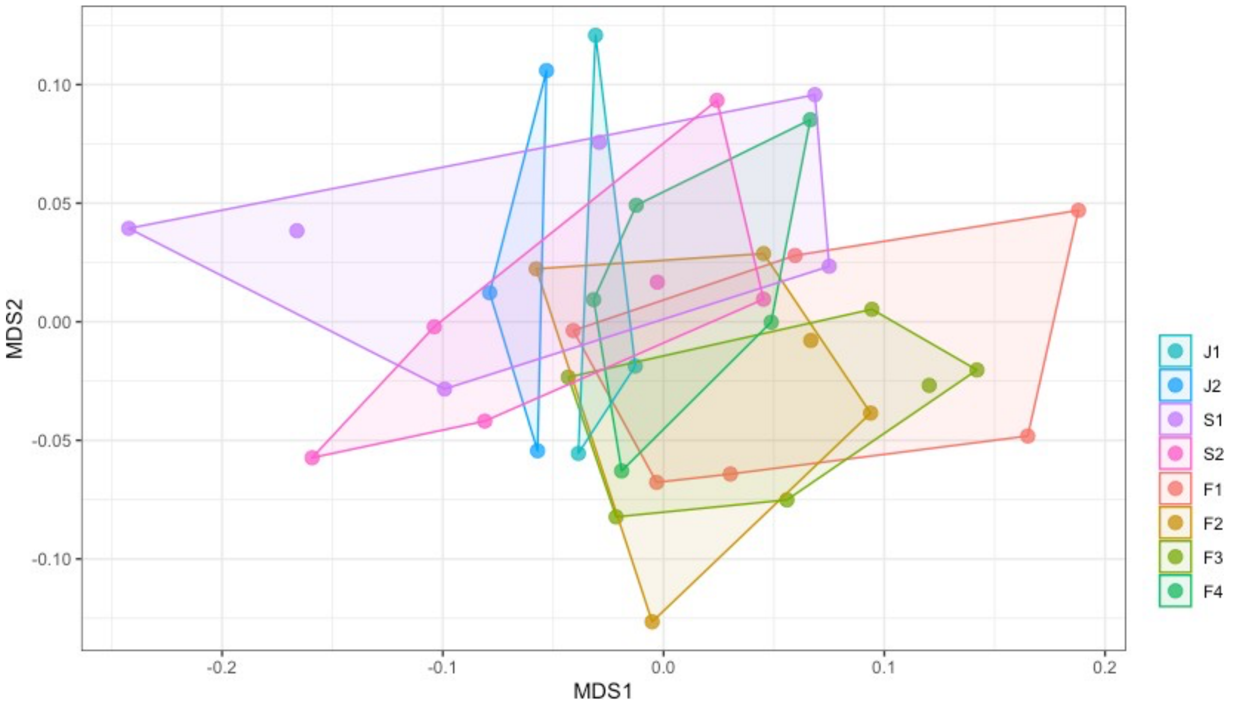

Figure S2. Multidimensional scaling (MDS) plot comparing Bray-Curtis dissimilarity across n=8 female southern white rhinoceros at the North Carolina Zoo sampled between 2020-2021.

Table S1. Summary of samples collected and datapoints used for age and seasonality statistical comparisons across n=8 female southern white rhinoceros at the North Carolina Zoo between July-September 2020 and January-March 2021.

Individual	Summer Collection Period			Winter Collection Period		
	**July**	**August**	**September**	**January**	**February**	**March**
J1	No	No	No	Yes^{A}	Yes^{A}	Yes^{A,S}
J2	No	No	No	Yes^{A}	Yes^{A}	Yes^{A,S}
S1	Yes^{S}	Yes^{S}	Yes^{S}	Yes^{A,S}	Yes^{A,S}	Yes^{A,S}
S2	Yes^{S}	Yes^{S}	Yes^{S}	Yes^{A,S}	Yes^{A,S}	Yes^{A,S}
F1	Yes^{S}	Yes^{S}	Yes^{S}	Yes^{A,S}	Yes^{A,S}	Yes^{A,S}
F2	Yes^{S}	Yes^{S}	Yes^{S}	Yes^{A,S}	Yes*	Yes^{A,S}
F3	Yes^{S}	Yes^{S}	Yes^{S}	Yes^{A,S}	Yes^{A,S}	Yes^{A,S}
F4	Yes^{S}	Yes^{S}	Yes^{S}	Yes^{A,S}	Yes^{A,S}	No

*Datapoint fell under rarefaction threshold (<36,100 reads) and was filtered out of dataset
^{A}Datapoint used for age-related statistical comparisons.
^{S}Datapoint used for seasonality statistical comparisons.
